# The Omega-3 Fatty Acid Eicosapentaenoic Acid Accelerates Disease Progression in a Model of Amyotrophic Lateral Sclerosis

**DOI:** 10.1371/journal.pone.0061626

**Published:** 2013-04-19

**Authors:** Ping K. Yip, Chiara Pizzasegola, Stacy Gladman, Maria Luigia Biggio, Marianna Marino, Maduka Jayasinghe, Farhan Ullah, Simon C. Dyall, Andrea Malaspina, Caterina Bendotti, Adina Michael-Titus

**Affiliations:** 1 Blizard Institute, Queen Mary University of London, London, United Kingdom; 2 Department Neuroscience, Istituto di Ricerche Farmacologiche Mario Negri-IRCCS, Milano, Italy; 3 Department of Life Sciences, University of Roehampton, London, United Kingdom; 4 North-East London and Essex MND Care Centre, Royal London Hospital, London, United Kingdom; Julius-Maximilians-Universität Würzburg, Germany

## Abstract

Amyotrophic lateral sclerosis (ALS) is a progressive fatal neurodegenerative disease characterised by loss of motor neurons that currently has no cure. Omega-3 polyunsaturated fatty acids, such as eicosapentaenoic acid (EPA), have many health benefits including neuroprotective and myoprotective potential. We tested the hypothesis that a high level of dietary EPA could exert beneficial effects in ALS. The dietary exposure to EPA (300 mg/kg/day) in a well-established mouse model of ALS expressing the G93A superoxide dismutase 1 (SOD1) mutation was initiated at a pre-symptomatic or symptomatic stage, and the disease progression was monitored until the end stage. Daily dietary EPA exposure initiated at the disease onset did not significantly alter disease presentation and progression. In contrast, EPA treatment initiated at the pre-symptomatic stage induced a significantly shorter lifespan. In a separate group of animals sacrificed before the end stage, the tissue analysis showed that the vacuolisation detected in G93A-SOD1 mice was significantly increased by exposure to EPA. Although EPA did not alter motor neurone loss, EPA reversed the significant increase in activated microglia and the astrocytic activation seen in G93A-SOD1 mice. The microglia in the spinal cord of G93A-SOD1 mice treated with EPA showed a significant increase in 4-hydroxy-2-hexenal, a highly toxic aldehydic oxidation product of omega-3 fatty acids. These data show that dietary EPA supplementation in ALS has the potential to worsen the condition and accelerate the disease progression. This suggests that great caution should be exerted when considering dietary omega-3 fatty acid supplements in ALS patients.

## Introduction

Amyotrophic lateral sclerosis (ALS) is the most common form of motor neuron disease. It is a neurodegenerative disease associated with loss of motor neurons in the spinal cord, brain stem and motor cortex, resulting in significant disability which ultimately leads to paralysis and death [1]. The median survival is 3–5 years from the onset of symptoms [2]. Currently, there is no cure for ALS and the only drug currently available is riluzole, which can only extend the lifespan of ALS patients by a few months [3]. Therefore, there is the uttermost need to find more effective therapies that will be beneficial to ALS patients.

Omega-3 polyunsaturated fatty acids (PUFAs) are natural compounds which have been associated with significant health benefits [4]. Furthermore, our laboratory and others have reported data which suggest that the long-chain omega-3 PUFAs eicosapentaenoic acid (EPA) and docosahexaenoic acid (DHA) have significant neuroprotective potential [5], . In aged rat brain, dietary supplementation with a mixture of EPA and DHA reverses age-related decreases in nuclear receptors and increases neurogenesis [8]. The neuroprotective effects of EPA and DHA can potentially occur through a variety of mechanisms, including a reduction in oxidative stress, excitotoxicity and neuroinflammation, and activation of anti-apoptotic pathways [9], [10], [11], processes which are very relevant in the context of the pathophysiology of ALS [12].

It is interesting to note that abnormalities in lipids have been linked with ALS. A number of studies have shown hyperlipidaemia to be a typical feature of ALS, and also positively correlated with disease progression and survival [13], [14]. Furthermore, a recent report showed that a high-fat diet in transgenic mice partially reversed the expression of markers of muscle denervation, delayed motor neuron death and also extended the lifespan of mice by 20% [15].

The aim of the present study was to explore the potential neuroprotective effects of EPA in a transgenic SOD1 mouse model of ALS. The G93A-SOD1 mice, that overexpress the human mutant SOD1 (Gly-93-Ala substitution) gene, exhibit pathological features that are very similar to human ALS [16], [17], and have been previously used in our laboratories [18], [19]. We investigated both the effect of treatment initiated at the disease onset or at a pre-symptomatic stage. We chose a daily dose of EPA of 300 mg/kg, which is in the range of doses of omega-3 PUFA reported to be neuroprotective for the central nervous system [5], [8]. The effects of the treatment were investigated using behavioral and histological endpoints, and also in terms of impact on disease onset and evolution, and survival.

## Materials and Methods

### Ethical statement

Procedures involving animals and their care were conducted according to the Mario Negri's institutional guidelines, that are in compliance with national (D.L. no. 116, G.U. suppl. 40, Feb. 18, 1992, Circular No.8, G.U., 14 luglio 1994) and international laws and policies (EEC Council Directive 86/609, OJ L 358, 1 Dec.12, 1987; NIH Guide for the Care and use of Laboratory Animals, U.S. National Research Council, 1996). All the experiments and the protocol proposed in the projects were examined first by Institutional Ethical Committee and then sent to the Italian Ministry of Health for authorization. The mice were bred and maintained in a SPF environment. Animals with substantial motor impairment had food on the cage bottom and water bottles with long drinking spouts. The survival time was defined as the time when the animals were unable to right themselves within 30 s after being placed on either side. The animals were deeply anesthetized with Equithesin and then sacrificed by decapitation before proceeding to the dissection of tissues or to undergo intracardiac perfusion if tissues were needed for use in immunohistochemistry.

### Animals and treatment

Female adult transgenic G93A-SOD1 mice on a C57BL/6JOlaHsd (C57/G93A) or a 129S2/SvHsd (129Sv/G93A) genetic background and their corresponding non-transgenic littermates were used in this study. The two G93A-SOD1 strains differ in the disease onset and duration [20]. Male mice are normally used for breeding to maintain the colony. All mice were maintained at a temperature of 21±1°C with a relative humidity of 55±10% and 12 h of light, and food and water were available *ad libitum.*

Animals were fed either a standard rodent powdered diet (control diet) or a diet supplemented with the EPA-enriched oil Incromega SE7010R (Croda Healthcare, U.K.) which provided an average intake of 300 mg/kg/day EPA (EPA diet). Incromega SE7010R contains 70% EPA and around 10% DHA, both in an ethyl ester forms, and the rest represented by other components. The food was replaced daily. Mice were housed in cages (n = 3–4 per cage) which contained a purpose built plastic box containing 4 holes for powdered food access, with minimal food spillage. The amount of food eaten for each cage was recorded twice weekly and the data divided for the number of mice present in the cage. In a pilot bioavailability study we assessed whether the addition of EPA-oil to the diet or the oral gavage once a day with EPA-enriched oil to provide 300 mg/kg, led to comparable changes in plasma fatty acids (n = 5 animals per group). At the end of 7 days exposure to EPA, animals were killed and the plasma collected and frozen until lipid analysis.

In one experiment C57BL/6 G93A-SOD1 mice (n = 16 each group) received EPA-enriched or control diet daily starting at disease onset (16 weeks age). In another experiment both G93A-SOD1 mouse strains (n = 14 animals each group) were treated daily with control or EPA-enriched diet, starting at a presymptomatic stage (12 weeks age for the 129Sv strain and 14 weeks age for the C57BL/6 strain).

### Disease progression assessment

Disease progression assessment was performed three times a week from the start of the experiment, by an operator blinded to the treatment. The parameters measured included body weight, locomotor activity on the Rotarod, grip strength and the hindlimb extension reflex as previously described [20]. The disease onset was determined by 1) a decrease in 3–5% body weight in comparison to the animal's maximum weight, 2) a reduction in the hind limb extension reflex when lifted by the tail, and 3) a decrease of around 10% in maximal Rotarod performance and/or grip strength. The survival time was defined as the time when the animals were unable to right themselves within 30 s after being placed on either side.

### Lipid analysis

The plasma and the spinal cord and brain tissue were harvested and frozen at −80°C until analysis. The plasma fatty acids were measured by the Nutrition Analytical Service (University of Stirling, Scotland). The tissue lipid analysis was carried out as previously described [21]. Lipids were extracted using the method of [22] with 0.01% w/v 2,6-di-tert-butyl-*p*-cresol (butylated hydroxytoluene, BHT) added as an antioxidant, and total phospholipids were then isolated by thin layer chromatography [23]. The fatty acid composition of phospholipids was measured after transesterification with 14% boron trifluoride in methanol and individual fatty acids identified by gas chromatography coupled to mass spectrometry (Agilent 6890 gas chromatograph connected to an Agilent 5973 mass selective detector, Agilent technologies, U.S.A.) using a Supelcowax 10 capillary column (30 m×0.25 mm×0.25 mm), as described previously [21]. Lipid identity was confirmed by retention times compared to known standards and mass spectra comparison to the National Institute of Standards and Technology database. Quantification was performed on selected ion peak area by ChemStation software (Agilent Technologies, U.S.A). Corrections were made for variations in the detector response and values of detected fatty acids were normalised to 100% and expressed as mol%.

### Histology

For the histological analysis, four groups of 4 mice each were treated from 14 weeks of age for 6–7 weeks with the following regime: 1) Non-transgenic wild type mice on control diet, 2) G93A-SOD1 mice on control diet, 3) Non-transgenic wild type mice on EPA diet, and 4) G93A-SOD1 mice on EPA diet.

When the G93A-SOD1 mice on the control diet exhibited a 50% reduction in the Rotarod performance and body weight loss, all the mice, including those on the EPA diet, were sacrificed and perfused with 4% paraformaldehyde, and the tissues were post-fixed as previously described [20].

The spinal cords were allocated into four batches, each containing one animal from each treatment group. The lumbar spinal cord segments were cut into 15 µm cryostat sections, stained and analysed by a blinded operator. Sections were allowed to dry for at least an hour before storage at −80°C until further processing.

- **Histochemistry.** To analyze the gross structural morphology, the spinal cord sections were stained for haematoxylin and eosin (H&E) using a standard protocol. Briefly, slides (stained together in batches containing tissue from an animal from each treatment group) were removed from −80°C and allowed to defrost for 5 min before a wash in tap water for 30 s then incubation in haematoxylin (700 µl/slide) for 2 min. Sections were washed in tap water for 30 s, differentiated in acid alcohol for 2 s then washed again in tap water for 30 s. After incubation with 2% calcium bicarbonate for 2 s and a wash in tap water for 30 s, sections were incubated in 1% eosin for 30 s, followed by 30 s wash in tap water. Sections were dehydrated in ascending alcohol concentration for 30 s each, cleared in 2 changes of Histoclear for 1 min each, then mounted with Histomount. Images were captured using a Zeiss Axioskop microscope and the Axiovision program.
- **Immunohistochemistry.** To analyze for cellular changes, the spinal cord sections were immunostained with the following antibodies: NeuN (neuronal marker, Chemicon, 1∶500), GFAP (astrocytic marker, Dako, 1∶1000), Iba1 (activated and non-activated microglial marker, Wako, 1∶1000), OX42 (activated microglial marker, Serotec, 1∶500), ChAT (motor neuron marker, Chemicon, 1∶100), 4-hydroxy-hexenal (4-HHE) (lipid oxidation marker, JaICA, 1∶100).

Sections (each batch of staining containing tissue from one animal from each treatment group) were removed from −80°C and allowed to defrost for 5 min. After 3×5 min washes in phosphate buffered saline (PBS), sections were incubated overnight in the chosen antibodies at room temperature. The next day, sections were washed 3×5 min in PBS then incubated in the secondary antibodies (donkey anti-goat, -mouse, -rabbit Alexa Fluor 488 or 568; final concentration 1∶1000) for 2.5 h at room temperature. After 3×5 min washes in PBS, sections were coverslipped in Vectashield containing the DAPI stain, to visualize the nuclei. Fluorescent Nissl staining of spinal cord sections involved incubation with NeuroTrace green-fluorescent Nissl stain for 20 min (N-21480, Molecular Probes, Invitrogen; 1∶100) [24]. Six to eight sections per animal were captured on a Zeiss microscope using the Axiovision v4.6 program. To avoid capture variability, each batch of immunostaining was captured in the same session using the same settings.

### Histological quantitative analysis

Six to eight sections per animal per group were analyzed in blind (to either treatment or disease) using Axiovision v4.8. For the analysis of vacuolization in the H&E staining set, a 500 µm×500 µm square (250,000 µm^2^) was placed over both the left and right dorsal and ventral horns. The areas of vacuolisation were recorded, and the left and right sides were averaged per section, with the data plotted as percentage of the 250,000 µm^2^ of area analyzed per one side of the spinal cord. In the analysis of intensity in immunostained sections, a 100 µm×100 µm square (10,000 µm^2^) was placed over the ventral horn. The number of cells with positive immunostaining and positive DAPI stained nuclei was recorded. The values from the left and right sides were averaged per section, with the data plotted as average intensity or number of positive cells per 10,000 µm^2^ of area analyzed per one side of the spinal cord. In the analysis of 4-HHE staining, microglia (i.e. Iba1 immunopositive cells) were drawn around (30 cells in the ventral horn) using the Axiovision program, which calculated the intensity of the 4-HHE immunostaining. Ten non-immunopositive areas directly adjacent to the Iba1 immunopositive cells were drawn around, to provide the background staining signal and thus provide a value for normalization purposes.

### Measurement of nitrotyrosine

The measurement of nitrotyrosine (NT) levels was carried out according to the protocol previously described [25]. Briefly, aliquots (2 µg protein) of total spinal cord homogenates from G93A-SOD1 mice and non transgenic littermates fed with control or EPA diet were loaded on nitrocellulose membrane, Trans-Blot Transfer Medium (Bio-Rad), by vacuum deposition on the Bio-Dot SF blotting apparatus (Bio-Rad). Membranes were probed over-night with the anti-3-NT monoclonal antibody (clone HM.11; Hycult Biotechnology) diluted 1∶1,000 and then with anti-mouse peroxidase-conjugated secondary antibody (Santa Cruz Biotechnology). Blots were developed by the ECL technique (homemade) and densitometric analysis of autoradiographic bands was carried out using a computer-assisted image analysis system (QuantityOne, BioRad). Immunoreactivity was normalized to the actual amount of protein loaded on the membrane as detected after Red Ponceau staining.

### Statistical analysis

All the data were plotted on graphs or in Table as means ± S.E.M. All the behavioural data were statistically analysed with GraphPad Prism (v5.03) software (GraphPad Software, Inc.) and all histological data were statistically analysed with SigmaStat (v3.5) software (Systat Software Inc.). The statistical significance for survival, disease onset and duration data was analyzed with the Gehan-Breslow-Wilcoxon test. The statistical significance for the other behavioral and biochemical data and the immunostaining was analyzed by two way single or repeated-measures ANOVA followed by Tukey's or Bonferroni's post-hoc test.

## Results

### 1) Increased plasma EPA content after oral gavage or dietary supplementation with EPA

In a first part of the study, focused on the bioavailability of EPA, we determined the level of EPA in plasma following free dietary intake, which provided a less invasive mode of administration, as compared to oral gavage. The dietary intake of EPA for 7 days by G93A-SOD1 C57BL/6 mice or 129Sv mice increased the plasma levels of EPA by 6.7 or 5.8 fold, respectively, when compared to the control diet (Table 1). An increase of 6.3 fold in plasma EPA was detected when G93A-SOD1 C57BL/6 mice received a single daily bolus of EPA at 300 mg/kg body weight by gavage and a smaller increase (3.8 fold) was seen after gavage in 129Sv mice (Table 1). Furthermore, the increases in plasma DHA seen after free dietary intake were also comparable (even slightly higher) than the increases seen after gavage, in the two strains. For example, the increase in C57BL/6 mice was 2.2 vs. 1.7 fold after dietary intake and gavage, respectively. The values in 129Sv mice were 1.7 and 1.3, respectively. This confirmed the adequate bioavailability of EPA using free dietary intake.

**Table 1:** Oral administration of EPA increases plasma EPA levels in G93A-SOD1 mice.

					Fold change	
Mouse strain	Fatty acid (% of total)	Control diet	EPA gavage	EPA diet	EPA gavage	EPA diet
C57BL6 mice	EPA	0.3±0.1	1.9±0.2 *	2.0±0.1 *	+6.3	+6.7
	DHA	3.1±0.9	5.3±0.4 *	6.8±0.6 *	+1.7	+2.2
	DHA/EPA	10.9±2.5	2.8±0.4 *	3.4±0.4 *	−3.9	−3.2
	Total n-3 PUFA	45.0±2.0	44.2±2.3	43.4±1.8	+1.0	+1.0
	Total n-6 PUFA	45.0±2.0	44.2±2.3	43.4±1.8	+1.0	+1.0
	Total PUFA	49.0±2.9	52.6±2.5	53.1±2.2	+1.1	+1.1
129Sv mice	EPA	0.4±0.1	1.5±0.5 *	2.3±0.3 *	+3.8	+5.8
	DHA	3.7±1.3	4.9±1.3 *	6.3±0.7 *	+1.3	+1.7
	DHA/EPA	9.0±2.4	3.6±1.6 *	2.8±0.2 *	−2.5	−3.2
	Total n-3 PUFA	4.8±1.6	7.3±1.4 *	9.3±1.0 *	+1.5	+1.9
	Total n-6 PUFA	46.5±3.7	48.5±1.7	46.8±0.5	+1.0	+1.0
	Total PUFA	51.3±4.5	55.8±2.7	56.1±1.3	+1.1	+1.1

Mice administered with either a single bolus dose of EPA (300 mg/kg, p.o.) or fed with an EPA enriched diet (300 mg/kg/day) for 7 days had higher levels of EPA and DHA in their plasma than mice fed on the control diet.

### 2) Dietary EPA administered at disease onset did not affect the course of motor deficit nor the survival length

**Figure 1:**
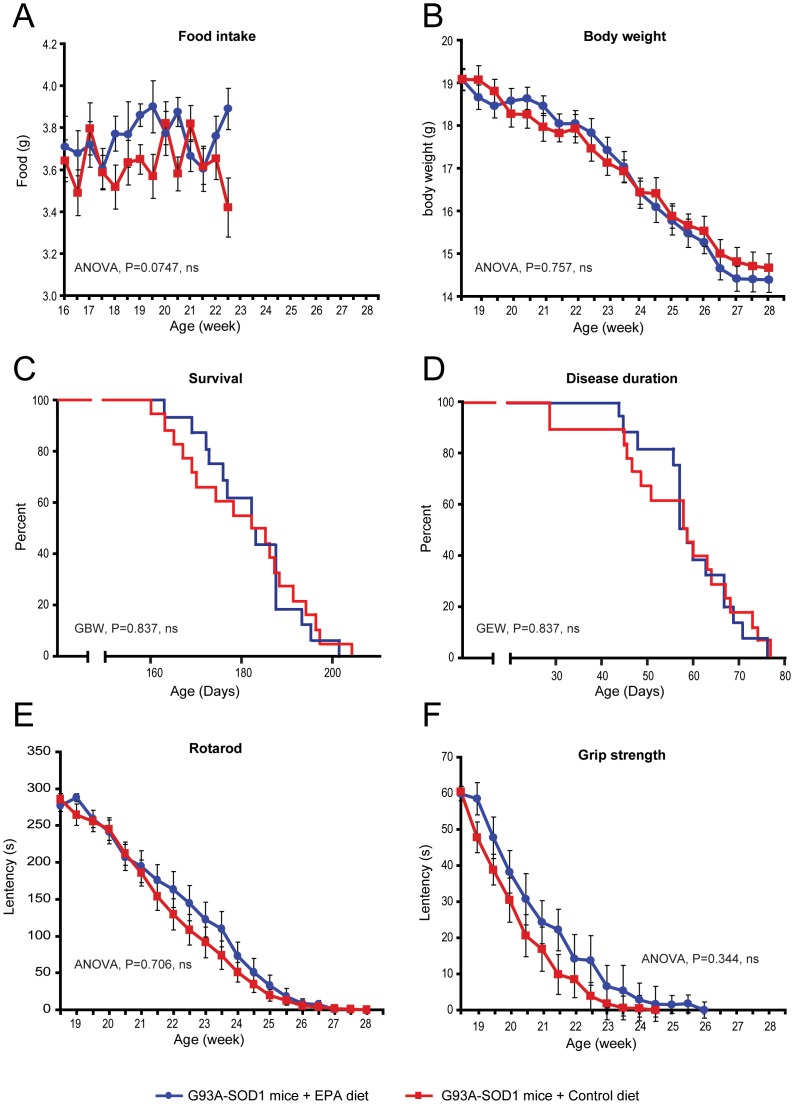
G93A-SOD1 C57BL/6 mice with dietary EPA at the symptomatic stage of the disease do not have a significantly different development of the disease compared to animals on the control diet. (A) Consumption of food is slightly increased when diet is enriched with EPA. (B) The body weight of mice was not affected as the disease evolves (C) Survival and (D) disease duration were not affected by dietary EPA. (E) The Rotarod and (F) The grip strength were not affected by dietary EPA.

In the first experiment, we initiated the treatment with EPA 300 mg/kg/day at disease onset, in G93A-SOD1 C57BL/6 mice. During treatment, there was a slight non-significant increase in food intake in the EPA diet group compared to the control diet group (Fig. 1A). However, there were no significant differences in the body weight loss during the development of the disease between the two treatment groups of G93A-SOD1 mice (Fig. 1B). Furthermore, no significant changes were observed in both survival and disease duration (Fig. 1C–D). There were no differences in the performance of the transgenic mice exposed to the control EPA diet, in the functional tasks of Rotarod and grip strength (Fig. 1E–F). Similar data were seen when the experiment was performed in 129Sv mice (Fig. S1). These observations indicated that in G93A-SOD1 mice, when dietary exposure to high EPA intake was initiated at the onset stage of the disease, the PUFA had no obvious impact on the disease process.

### 3) Dietary EPA intake at pre-symptomatic stage of ALS disease accelerates disease progression

**Figure 2:**
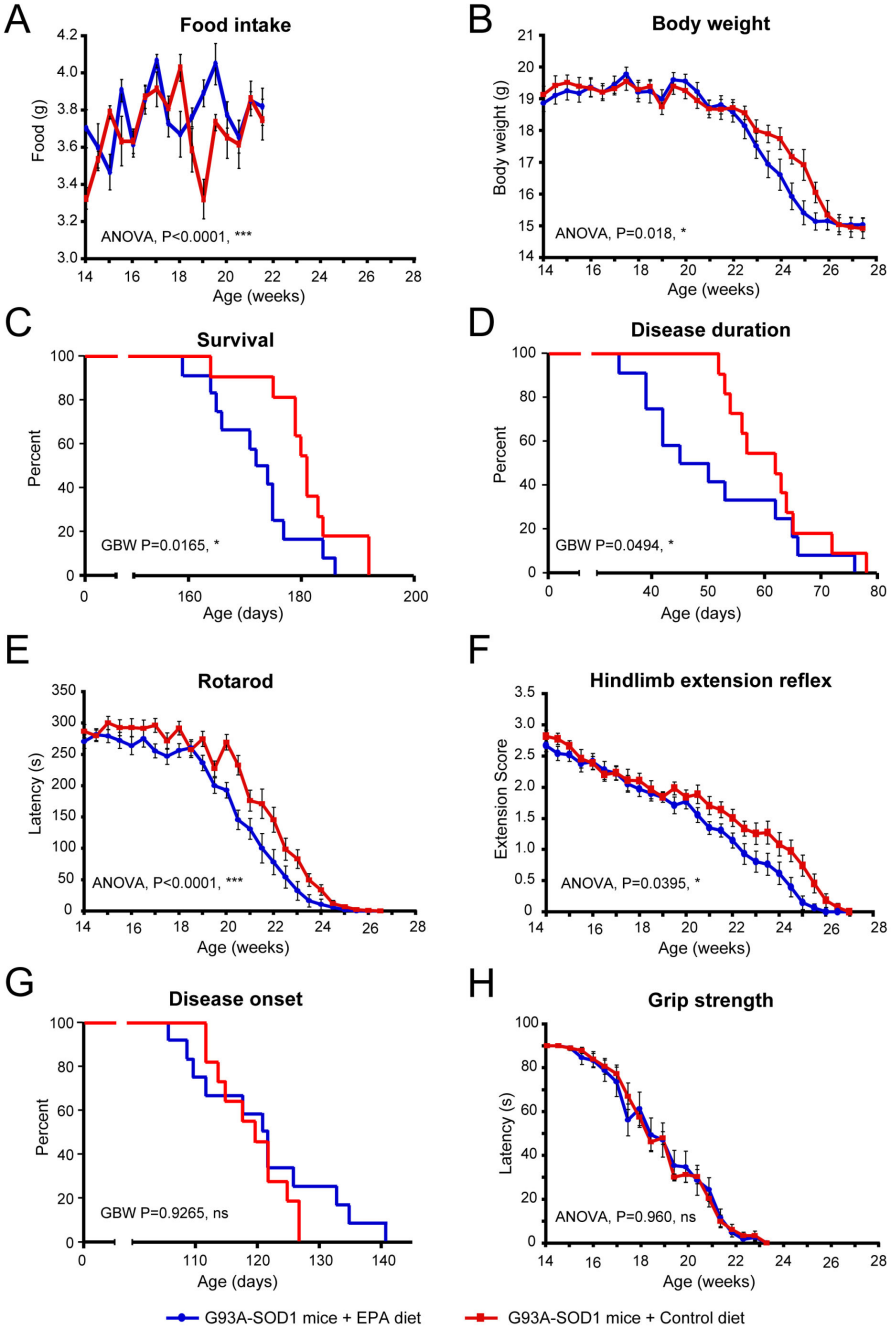
G93A-SOD1 C57BL/6 mice treated with dietary EPA at the pre-symptomatic stage of ALS have an accelerated development of the disease compared to G93A-SOD1 mice on the control diet. (A) Consumption of food is increased in animals on the EPA enriched diet. (B) The body weight of mice with EPA diet decreases as the disease evolves. (C) Survival and (D) disease duration are both reduced with dietary EPA. (E) The Rotarod and (F) Hindlimb extension reflex are both affected by dietary EPA. (G) The rate at which the disease starts is not affected by dietary EPA. (H) The grip strength was not affected by dietary EPA.

The lack of effects seen when the treatment was initiated at disease onset led us to hypothesize that a longer exposure, in the transgenic animals with the slower disease evolution, may provide a better opportunity to characterize a neuroprotective potential. Therefore, treatment was initiated at a pre-symptomatic stage (14 weeks of age). The amount of diet eaten by G93A-SOD1 mice when exposed to EPA enrichment at a pre-symptomatic stage of the disease was significantly greater overall, during the experiment, than by G93A-SOD1 mice on the standard control diet, although there was a certain variability (Fig. 2A). Therefore, EPA appeared to increase the appetite of the animals. However, unexpectedly, in spite of an increase in food intake, there was a significant higher body weight loss in G93A-SOD1 mice on the EPA diet compared to the control diet group (Fig. 2B).

The dietary intake of EPA (300 mg/kg/day) by G93A-SOD1 C57BL/6 mice significantly enhanced the rate of disease progression, indicated by parameters such as survival (loss of righting reflex) and disease duration (Fig. 2C–D). Furthermore, the functional tests on the Rotarod and the hind limb extension reflex showed a significant reduction in the animals' ability to use their hind limbs, when the EPA group was compared to the control diet group (Fig. 2E–F). Interestingly, no significant difference in disease onset (Fig. 2G) and grip strength (Fig. 2H) was seen between EPA treated and control animals. This data suggest that G93A-SOD1 mice at the pre-symptomatic stage of the disease when fed the EPA diet have an accelerated disease progression. A similar trend was observed in G93A-SOD1 129Sv mice treated with 300 mg/kg/day starting the treatment at pre-symptomatic stage (12 week age) (Fig. S2).

Therefore, quite unexpectedly, this indicated overall that the prolonged exposure to the EPA diet and the initiation of the exposure to this fatty acid at a pre-symptomatic stage, led to an accelerated disease progression. The lack of significant difference in the grip strength suggests that the EPA diet could influence the disease progression mostly in the hind limbs, which are more affected in respect to the forelimbs.

### 4) Dietary EPA is linked to intense vacuolisation

**Figure 3:**
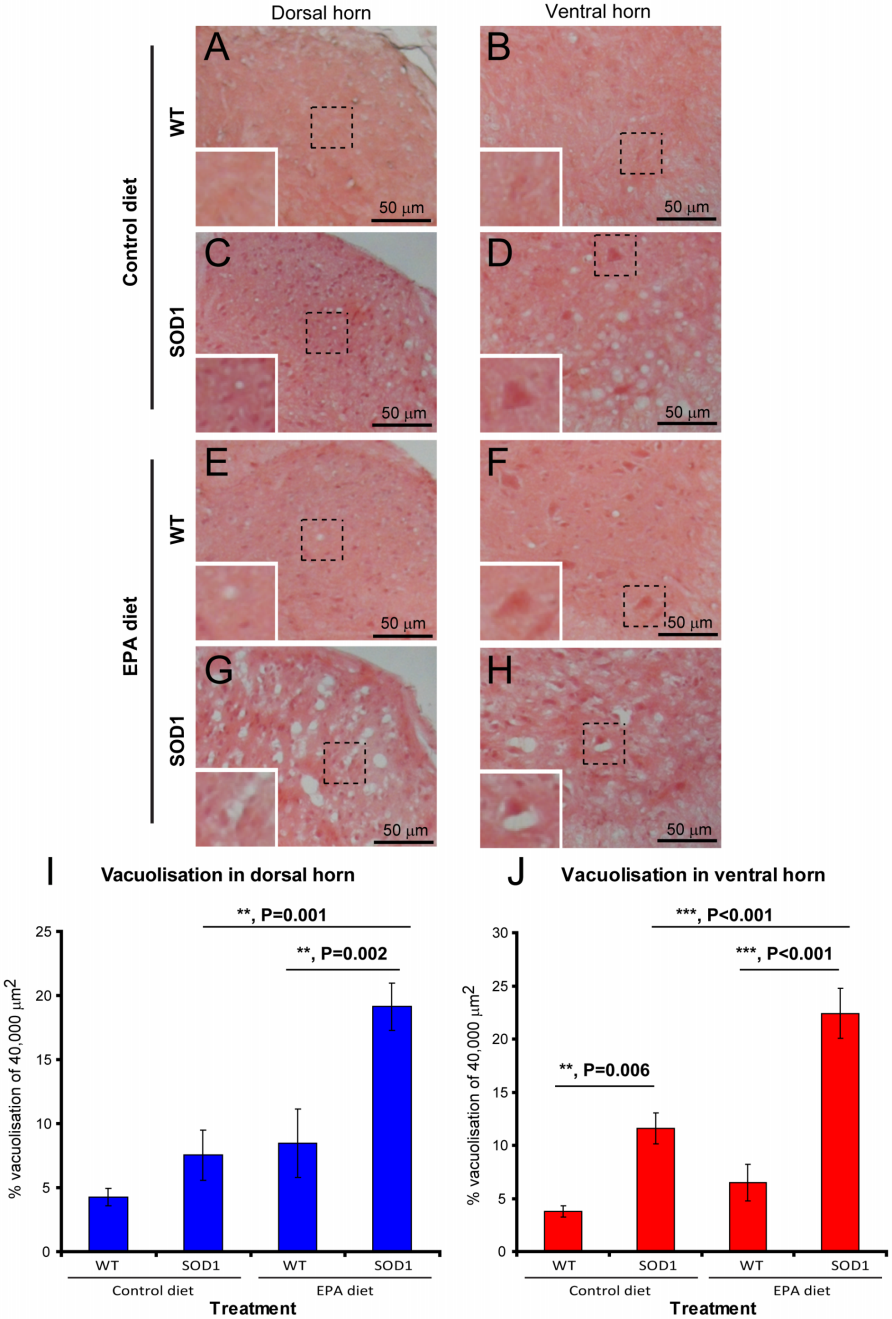
G93A-SOD1 C57BL/6 mice treated with EPA at the pre-symptomatic stage of the disease have an increase in vacuolization in the spinal cord. (A–B) Wild type littermates on the control diet have very sparse vacuoles within the spinal cord. (C–D) G93A-SOD1 mice on the control diet have vacuoles predominantly in the ventral horn. (E–F) Wild type littermates on the EPA diet have a slight increase in vacuoles within the spinal cord. (G–H) G93A-SOD1 mice on the EPA diet have a marked increase in vacuoles in the dorsal and ventral horn. (I–J) Quantitative analysis of vacuolisation in the spinal cord. Scale bar = 50 µm.

In order to identify the changes which may underlie the disease worsening seen after EPA exposure at the pre-symptomatic stage, described above, we treated G93A-SOD1 C57BL/6 animals for several weeks with EPA, but we sacrificed them before the terminal stage, for tissue analysis. First, H & E staining was carried out, to assess the crude morphology, and this revealed clearly visible vacuoles in the lumbar spinal cord of G93A-SOD1 mice compared to the wild-type littermate mice (Fig. 3A–H). Interestingly, in the control diet groups, the G93A-SOD1 mice only showed a significant increase in vacuolisation in the ventral horn and not in the dorsal horn, when compared with the wild-type littermate mice. (Fig. 3A–B). The analysis showed that there was a further marked and significant increase in vacuolisation within the dorsal and ventral horn of the G93A-SOD1 mice treated with the EPA diet, compared to the control diet (Fig. 3I–J). Furthermore, the cells appeared more intensely eosinophilic in the G93A-SOD1 mice EPA treatment compared to the other groups (Fig. 3C–D, G–H).

### 5) Dietary EPA is not associated with significant alterations in neuronal cells

**Figure 4:**
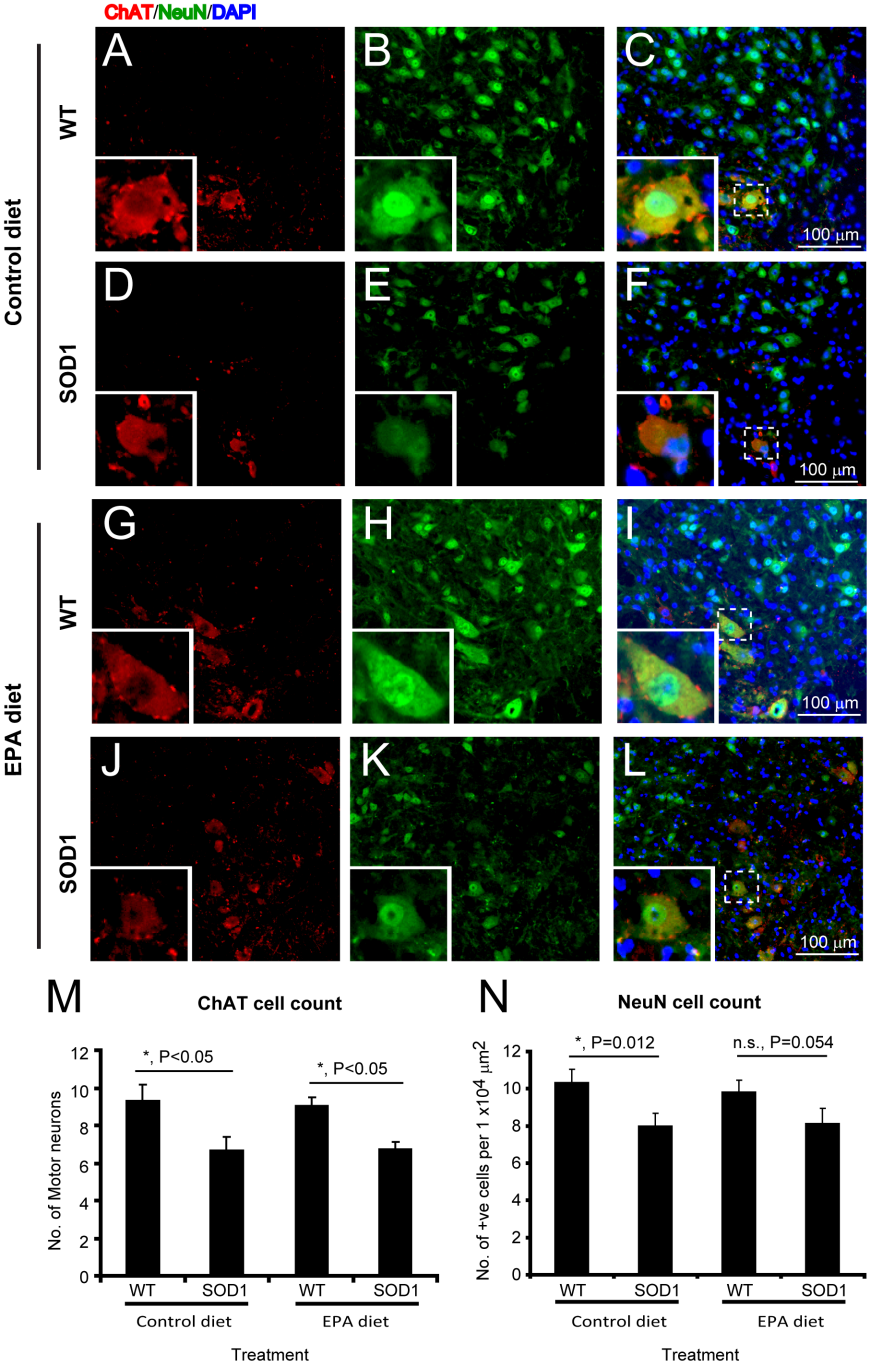
Neuronal loss in G93A-SOD1 C57BL/6 mice treated with EPA at the pre-symptomatic stage of the disease is not affected by treatment. The G93A-SOD1 mice have significantly fewer neurons compared to the control wild type littermates, when the tissue is labelled with ChAT (motor neuron marker, red) or NeuN (general neuronal marker, green) (A–L). Nuclei are stained with DAPI (blue). No significant difference in cell number was observed between the EPA and control diet groups (M). Scale bar = 100 µm.

To determine the neuronal changes in the spinal cord, immunohistochemistry and quantitative analysis was carried out as previously described [26]. Neurons and more specifically motor neurons were visualized with the immunomarkers NeuN and ChAT, respectively. There was a significant reduction in both NeuN and ChAT immunopositive cell counts in G93A-SOD1 C57BL/6 mice compared to wild type littermate controls (Fig. 4A–N). In the groups on dietary EPA, there was no significant difference in neuronal staining in either the wild type littermates or G93A-SOD1 mice groups when compared to the groups on the control diet (Fig. 4M–N).

**Figure 5:**
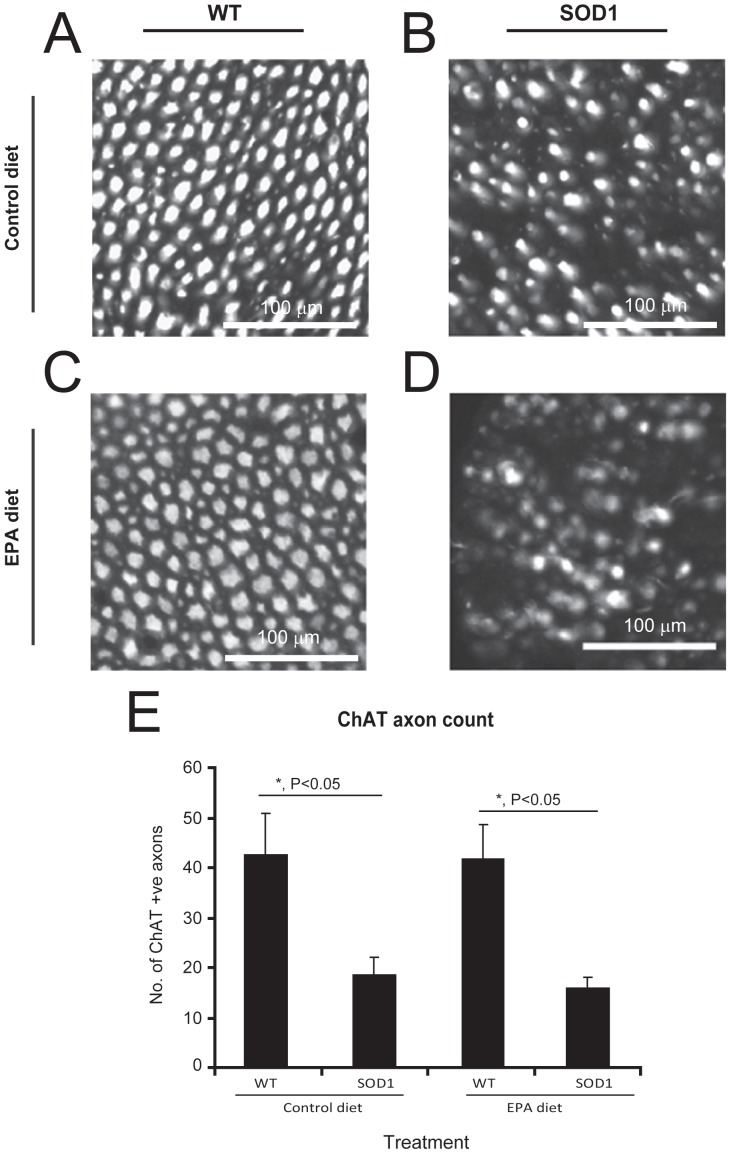
Motor neuron axonal loss in G93A-SOD1 C57BL/6 mice treated with EPA at the pre-symptomatic stage of the disease is not affected by dietary EPA. ChAT-labelled motor neuron axons can be observed (A–D). There are significantly fewer axons in G93A-SOD1 mice compared to the wild type littermates. No significant difference in the number of axons was observed between the EPA and control diet groups. Scale bar = 100 µm.

The axons of motor neurons in the ventral roots, which can be visualized using ChAT immunostaining, showed a significant decrease in the axon diameter size in the G93A-SOD1 mice compared to the wild type littermate mice, in both the control and EPA diet groups (Fig. 5A–E). However, there was no significant difference between G93A-SOD1 mice on either the EPA or the control diet (Fig. 5A–E).

### 6) Dietary EPA leads to alterations in glial cells

To determine if the non-neuronal cells, i.e. astrocytes and microglia, are involved in the deleterious effect of dietary EPA, immunostaining and quantitative analysis of non-neuronal cells was carried out.

**Figure 6:**
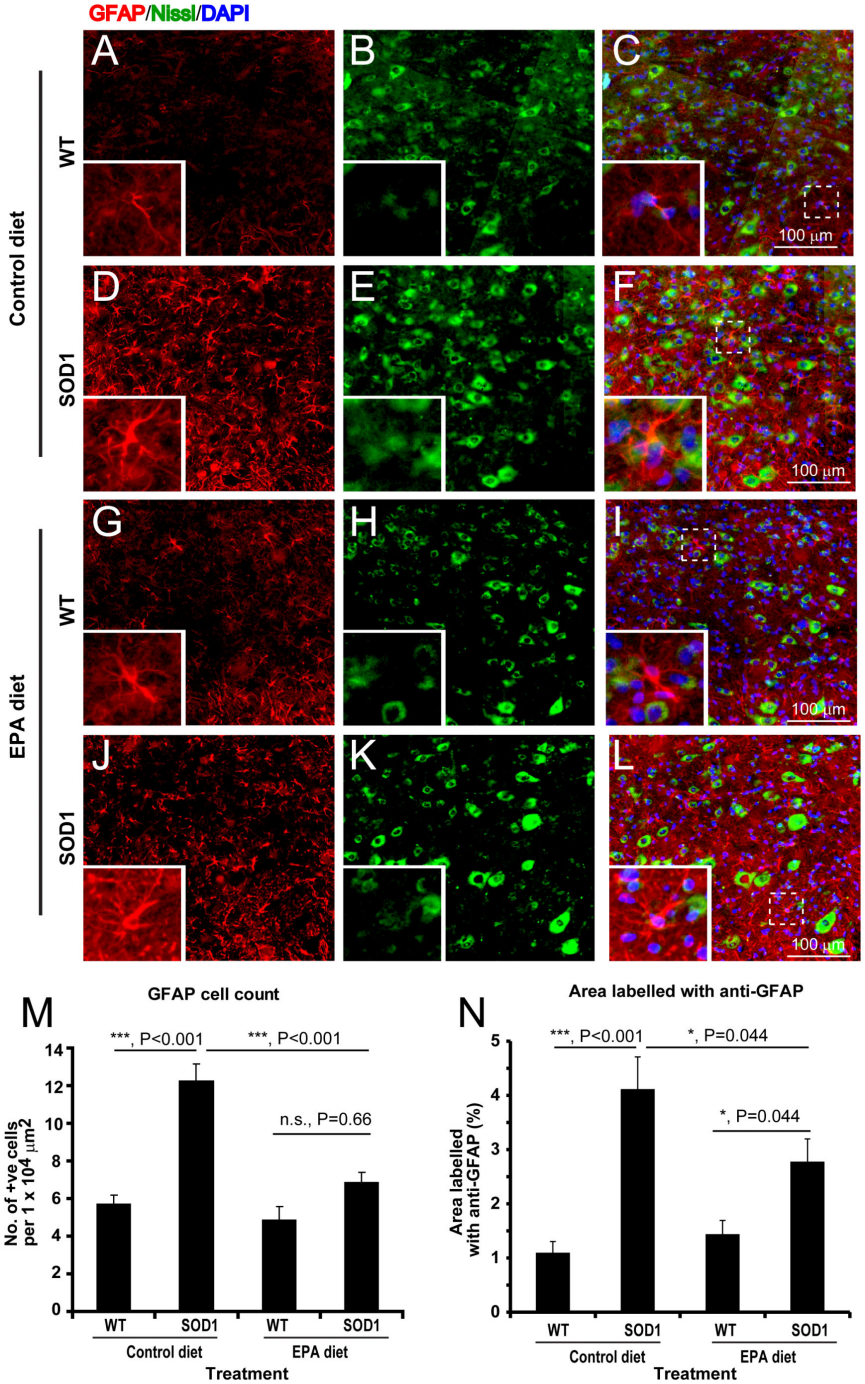
Astrocyte activation in G93A-SOD1 C57BL/6 mice treated with EPA at the pre-symptomatic stage of the disease is reduced by treatment. Some astrocytes can be observed in the wild-type littermate spinal cord (A–C). In the spinal cord of G93A-SOD1 mice, there is a significant increase in the number of astrocytes (D–F). The treatment with EPA did not significantly alter the number of astrocytes in wild type mice (G–I), but significantly reduced astrocytic activation in G93A-SOD1 mice (J–L) in comparison to the corresponding control group. Quantitative analysis of astrocytic cell number (M) and staining intensity (N) in the ventral horn of the mice spinal cord: GFAP (astrocytes, red), Nissl stain (neurons, green) and DAPI (nuclei, blue). Scale bar = 100 µm.

**Figure 7:**
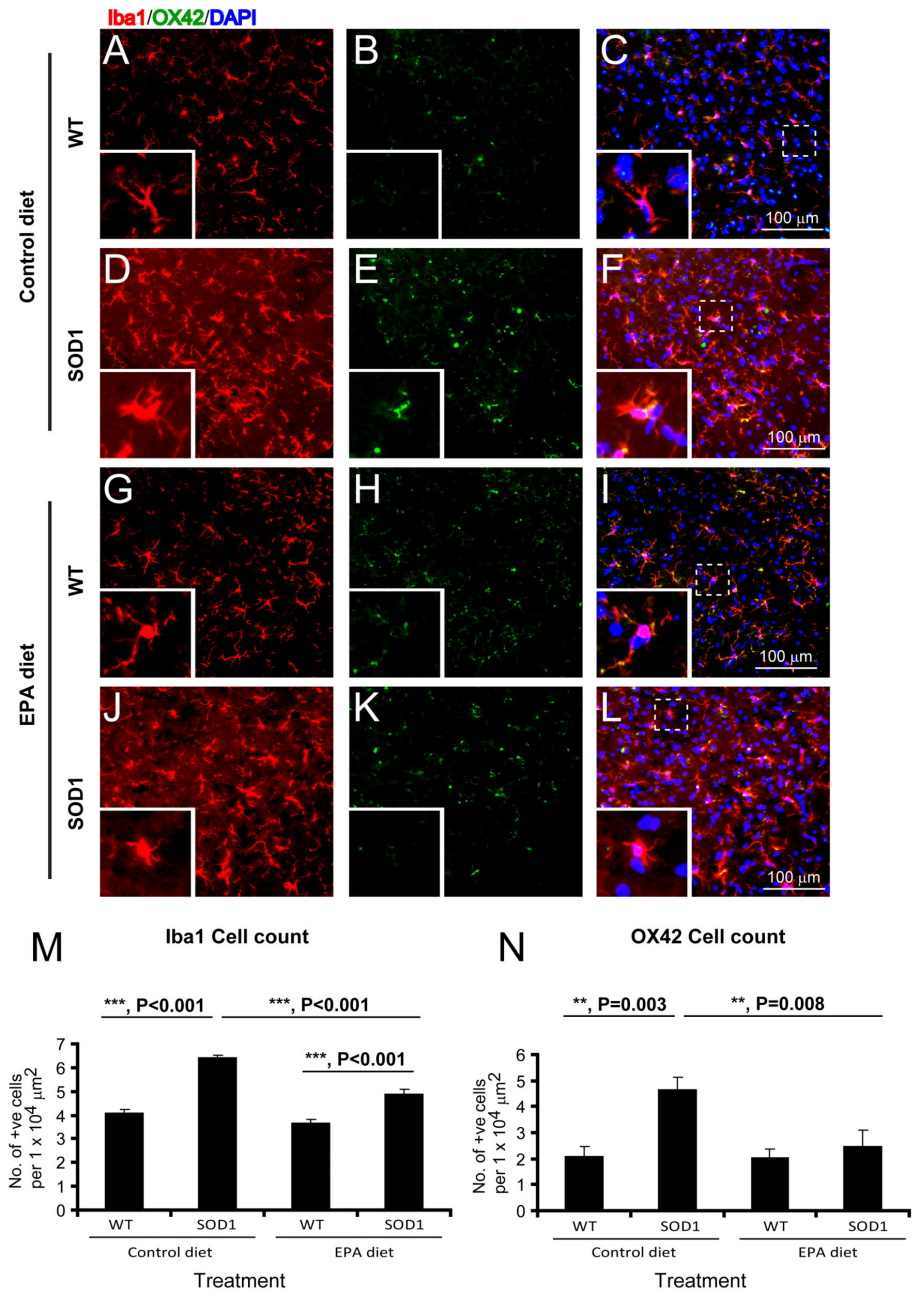
Microglial activation in G93A-SOD1 C57BL/6 mice treated with EPA at the pre-symptomatic stage of the disease is reduced by EPA. Microglia can be observed in the wild type littermate spinal cord with using Iba1 and OX42 labelling (A–C). In the spinal cord of G93A-SOD1 mice, there is a significant increase in the number of microglia (D–F). The treatment with EPA did not significantly alter the number of microglia in the wild type (G–I), significantly reduced it in G93A-SOD1 mice (J–L) in comparison to the corresponding group on the control diet. Quantitative analysis of microglia in the ventral horn of the spinal cord (M). Labelling shown is Iba1 (microglia, red), OX42 (activated microglia, green) and DAPI (nuclei, blue). Scale bar = 100 µm.

- **Astrocytes.** Astrocytes were visualized using GFAP as a marker, as previously [27]. There was a significant increase in the number and staining intensity of GFAP immunopositive cells present in the ventral horn of G93A-SOD1 mice compared to the wild-type littermates (Fig. 6A–F, M–N). However, after the EPA diet, the number and staining intensity of GFAP immunopositive cells in G93A-SOD1 mice was significantly lower than in the G93A-SOD1 mice on the control diet, with no statistically significant differences in comparison to the wild type littermates of either treatment groups (Fig. 6M–N).
- **Microglia.** Microglia were visualized using the immunomarkers Iba1 (activated and non-activated form) and OX42 (activated form) as previously [27]. There was a significant increase in the number of Iba1 and OX42 immunopositive cells in the ventral horn of G93A-SOD1 C57BL/6 mice compared to the wild type littermate mice, on the control diet (Fig. 7A–F, M). Interestingly, there was also a significant reduction in the number of Iba1 and OX42 immunopositive cells present in the ventral horn of G93A-SOD1 mice treated with the EPA diet compared to the G93A-SOD1 mice on the control diet (Fig. 7M–N).

These data suggested that the dietary intake of EPA could significantly reduce the levels of reactive astrogliosis and microglial reaction in the lumbar spinal cord of G93A-SOD1 mice.

### 7) Dietary EPA increases the lipid peroxidation in microglia

**Figure 8:**
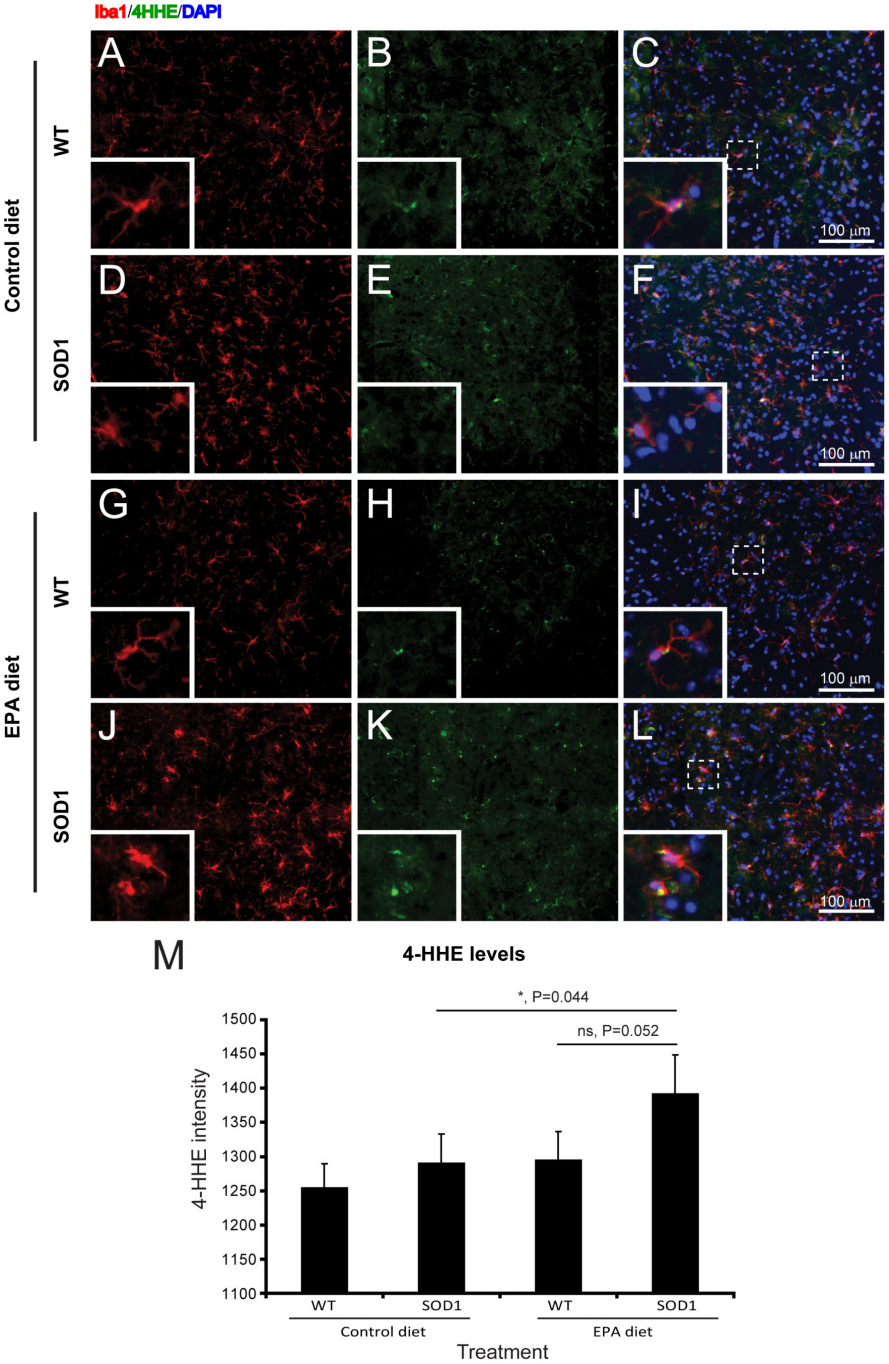
Microglia in G93A-SOD1 C57BL/6 mice treated with EPA at the pre-symptomatic stage of the disease have an elevated level of lipid oxidation. In the wild type littermates spinal cord (A–C) there is limited expression on 4-HHE. In the spinal cord of G93A-SOD1 mice, there was an increase in the level of 4-HHE expression (D–F). The treatment with EPA did not significantly alter the level of 4-HHE in the wild type mice (G–I), but significantly increased it in G93A-SOD1 mice (J–L) in comparison to the corresponding control group. Quantitative analysis of 4-HHE expression in the ventral horn of the spinal cord (M). Labelling shown is Iba1 (red), 4-HHE (green) and DAPI (blue). Scale bar = 100 µm.

To assess lipid-linked oxidation processes, the major lipid peroxidation product of omega-3 fatty acids, the immunostaining marker 4-hydroxy-2-hexenal (4-HHE) was used [28]. The expression of the oxidative marker 4-HHE was present at the highest level within the microglia, and not in neurons or astrocytes (data not shown). Within the microglia, the 4-HHE immunostaining level was significantly higher in the G93A-SOD1 mice on EPA diet compared to the control diet (Fig. 8A–M). Interestingly, the 4-HHE immunostaining level between the G93A-SOD1 mice and wild type littermates on the control diet was not significantly different (Fig. 8M).

### 8) Dietary EPA reduces the level of nitrotyrosine (NT) in lumbar spinal cord

**Figure 9:**
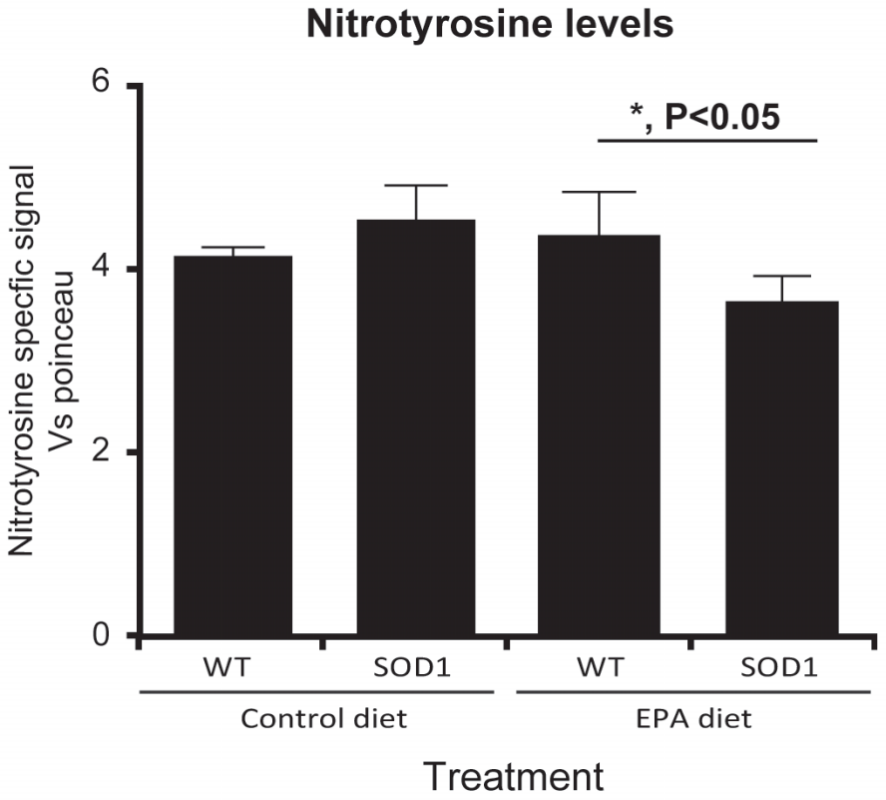
Decreased nitrotyrosine in G93A-SOD1 C57BL/6 mice treated with EPA at the pre-symptomatic stage of the disease. The level of nitrotyrosine was significantly reduced in the presence of dietary EPA compared to the control diet in G93A-SOD1 mice, but not in wild type littermates.

In order to examine whether the deleterious effect of dietary EPA could be due to an increase in protein oxidation, we determined the level of nitrotyrosine in the spinal cord of G93A-SOD1 mice fed with EPA from the pre-symptomatic stage in comparison to control standard diet fed mice. Although we did not observe a significant increase of nitrotyrosine in G93A-SOD1 mice compared to non-transgenic mice, the EPA diet significantly reduced the levels of NT in G93A-SOD1 mice in respect to control diet fed transgenic mice (Fig. 9).

### 9) Dietary EPA and tissue lipid content changes

**Figure 10:**
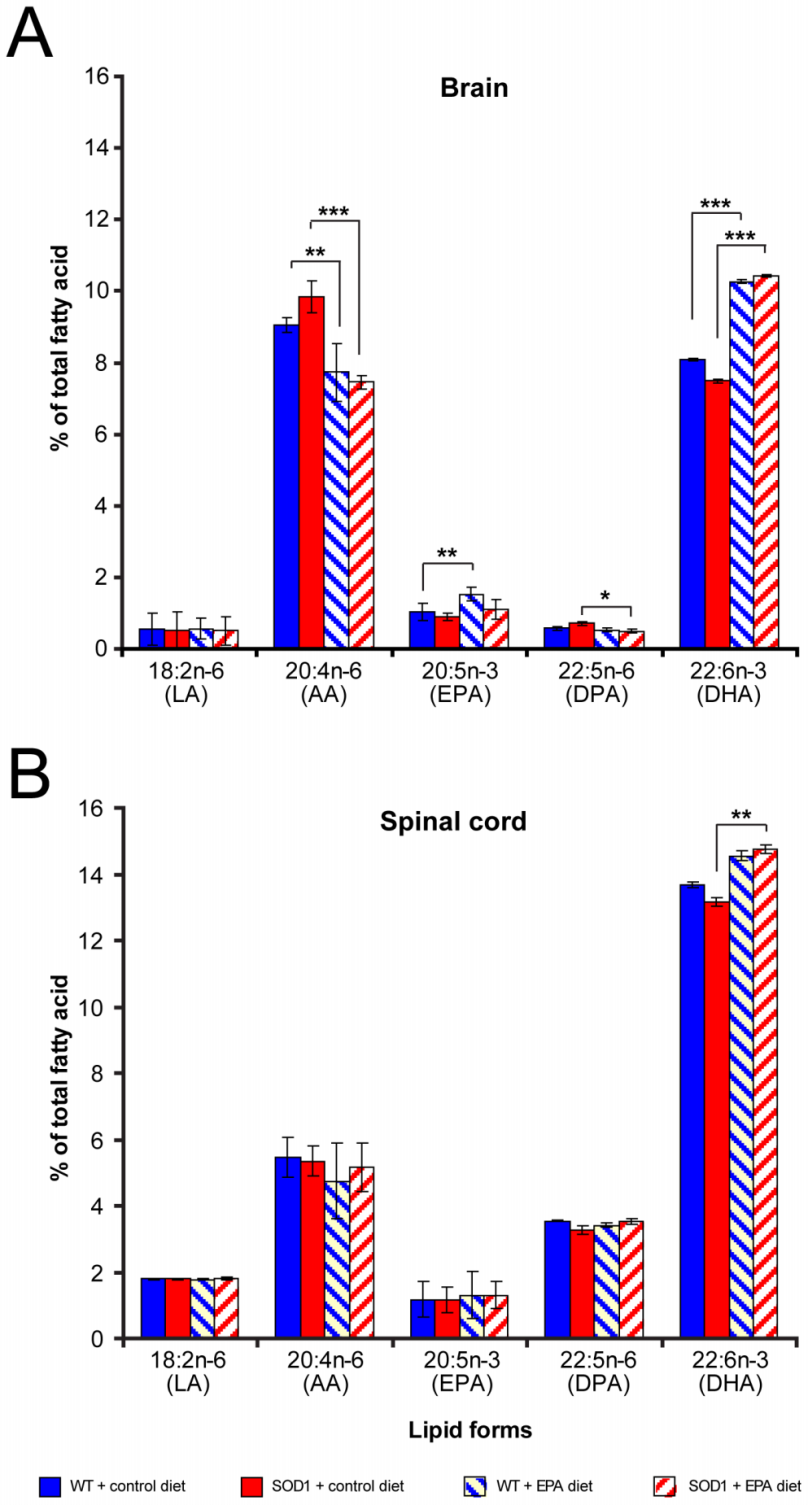
Increased EPA and DHA in the CNS of C57BL/6 mice treated with EPA at the pre-symptomatic stage of the disease. The level of DHA was significantly increased in both the brain (A) and spinal cord (B) in the presence of dietary EPA compared to the control diet in G93A-SOD1 mice.

To assess the impact of the EPA diet on the tissue lipid composition, we analyzed extracts of both spinal cord and brain from wild type and transgenic G93A-SOD1 mice that were exposed to the diet for the same period as the animals used for histological analysis. The results showed that the supplementation with EPA led to very modest changes in spinal cord tissue EPA and DHA (only a significant increase in DHA in transgenic animals). In contrast, the treatment led to more marked increases in EPA and DHA in brain tissue, in both wild type (EPA and DHA) and G93A-SOD1 mice (DHA only). There were corresponding decreases in arachidonic acid (AA) in both wild type and transgenic animals (Fig. 10).

## Discussion

The aim of the present study was to establish whether the long-chain omega-3 fatty acid EPA has a neuroprotective potential in ALS. The model chosen to test this was the extensively used G93A-SOD1 transgenic mouse model [18]. EPA either supplemented in the diet or as an oral bolus administration provided excellent bioavailability of the fatty acid. When the EPA-supplemented diet (a regime preferred for its non-invasive nature) was provided to animals at the disease onset, the treatment had no significant impact on disease onset and survival, nor did it improve neurological score. Unexpectedly, when dietary EPA was provided at a pre-symptomatic stage, the fatty acid decreased survival. Using histological and immunocytochemical analysis, the spinal cord tissue from G93A-SOD1 transgenic animals showed vacuolisation. However, the vacuolisation was amplified in the tissue of animals receiving EPA. A loss of neurons was also observed in the tissue of transgenic animals, but this loss was not exacerbated by dietary EPA, and neither was the significant motor neuron axonal loss, which was already apparent in transgenic animals. An analysis of early changes in neuronal synaptic markers would add more information on the impact of the treatment with EPA on the neurodegenerative mechanisms involved at an early stage of the disease. In accord with many reports in the literature, we found significant alterations in glial cells in G93A-SOD1 mice, supporting the concept of non-cell autonomous disease [29], [30], [31], [32]. Microglial activation and the appearance of reactive astrocytes have been described in ALS [11], [27], [28]. The expression of mutated SOD1 in astrocytes leads to neurotoxicity [33], [34], and astrocytes amplify pathogenetic processes, such as the oxidative stress. They up-regulate the production of nitric oxide, increasing nitrative stress. Interestingly, although in our study the nitrotyrosine production was not markedly affected by the disease, there was a small but significant reduction in the level of nitrotyrosine detected in the spinal cord of G93A-SOD1 mice receiving dietary EPA compared to the transgenic animals receiving the control diet. This effect could be explained by the reduced number of astrocytes observed in G93A-SOD1 mice on dietary EPA. Whether and how this could relate to the exacerbation of the disease symptoms is unknown. Immunostaining for a toxic lipid peroxidation marker specific for omega-3 fatty acids, 4-hydroxy-2-hexenal (4-HHE), showed a higher signal in microglia than in other cell types within the spinal cord, and was significantly higher in the EPA treated group compared to the other groups. Thus, chronic dietary EPA intake appears to increase the levels of one of the major toxic lipid peroxidation products, resulting in increased oxidative stress in microglia. The data clearly demonstrate that there is a detrimental rather than a protective effect of EPA on the progression of symptoms and the survival of G93A-SOD1 mice. How this effect mates with the reduction of astrocytes and microglia and the reduction of nitrotyrosine is not clear. We can only speculate as described below that the reduction of nitrotyrosine by EPA may be the consequence of the reduction of astrocytes. As concerns the microglia even if we observed a reduction in the cell number, those remained exhibit a high toxic lipid peroxidation which results in an increased oxidative stress with detrimental effect on the tissue vacuolization. This observation supports data reported by other authors, which documented significant lipid oxidation in the spinal cord of sporadic ALS patients, and in particular a high level of 4-HHE (in astrocytes, microglia and neurons) and a global increase in malondialdehyde [35], [36]. The study by Ilieva and collaborators (2007) showed a selective decrease in DHA in the spinal cord of patients, paralleled by an increase in the DHA content in the brain, which they interpreted as a possible endogenous neuroprotective attempt. We did not observe such changes in the tissue of our transgenic mice, but what was notable was the different response of the tissue to EPA supplementation: there were no notable changes overall in the spinal cord tissue after several weeks of dietary EPA intake, whereas there were more marked increases in the EPA and DHA levels in the brain tissue from the same animals. Overall, the lipid analysis showed, as expected, a predominant presence of DHA as a fatty acid tissue component, compared to EPA. It has been shown that this difference in brain tissue fatty acid levels may be due to the fact that EPA is subject to rapid β-oxidation [37].

Administration of EPA after compression spinal cord injury in rats has been shown to have significant neuroprotective effects on neurons in the ventral horn [7]. EPA treatment has also been shown to have beneficial effects in a mouse model of Huntington's disease, although the fatty acid only improved modestly motor dysfunction, without any parallel structural evidence of neuroprotection [38]. There are also reports documenting the protective effect of EPA in models of inflammation in the central nervous system [39], [40]. These observations led us to choose EPA for our first extensive studies (although there are beneficial central nervous system effects also reported for DHA) and hypothesize that dietary EPA may lead to some benefits in ALS. The long chain omega-3 fatty acids EPA and DHA are safe and well-tolerated even in high doses, and therefore could offer a large therapeutic window. Furthermore, these fatty acids are widely and freely available (most often as fish-oil capsules produced by various manufacturers), and based on the favorable lay reports and specialized literature on these compounds, ALS patients may resort to self-treatment with omega-3 fatty acids in an attempt to modify the disease, due to lack of effective therapies. The potential beneficial effects advertised by health groups are also supported by positive recommendations on these omega-3 fatty acids by the World Health Organization. The dose of 300 mg/kg/day used in this study was in the range of doses of DHA and EPA used in studies in rats and mice, and is equivalent to an adult human of an average weight of 70 kg receiving around 1.7 g of dietary EPA - if allometric scaling to human is used, in a manner similar to that recommended for drugs by the Food and Drug Administration (http://www.fda.gov/), and reflecting the difference in the mouse vs. human body surface area. Thus, the dietary EPA dose used in the present study is well within the dose ranges of omega-3 fatty acids used in several clinical studies, such as 24 weeks of 2.7 g EPA/day for Crohn's disease [41] to as high as 8 weeks of 13.5 g EPA/day for psoriatic lesion [42]. Furthermore, it has been recommended that athletes should consume up to 2 g EPA/day [43]. However, it must be noted that EPA in this study was provided in the form of the enriched Incromega oil, which contains 70% EPA and around 10% DHA, both in the ethyl ester forms, while the rest represented by other components. In comparison, several groups have shown the use of ultra-pure ethyl-EPA in clinical trials for Huntington's disease [44] Alzheimer's disease [45] and depression [46].

The detection of significantly increased vacuolisation in the G93A-SOD1 mice treated with the EPA diet compared to control diet suggests that there is a further increased cellular damage within the spinal cord when animals ingest this fatty acid. Vacuolisation is associated per se with the presence of aggregates of mutated SOD1 protein, which can occur before the onset of significant neuronal death [47]. Unsaturated fatty acids have been shown to promote aggregation of SOD1 mutants [48]. Therefore, it could be suggested that the EPA obtained from the diet slowly potentiated the aggregation of the mutated SOD1 protein and this could ultimately result in spinal cord mitochondrial damage. Spinal cord-specific factors appear to recruit SOD1 mutants to spinal mitochondria [49]. SOD1 enters the mitochondria in the demetallated form, and here it may encounter an increased oxidative stress. Increased oxidation is reflected in the higher level of 4-HHE detected for example in microglia in the present study. This oxidation may favour formation of small oligomeric aggregates, which may be the precursors of the toxic forms [50]. The mutant SOD1 localized in mitochondria could alter the expression of essential proteins, such as the anti-apoptotic bcl-2 [51], or mitochondrial voltage dependent ion channels [52].

The tissue vacuolisation may not be directly linked to neuronal cell loss in the first instance but may involve the non-neuronal cells such as astrocytes and microglia. Our data showed no significant changes in the numbers of neurons using NeuN or ChAT immunostaining, but a significant reduction in microglia (using Iba1 and OX42 immunostaining), and astrocytes (immunostained with GFAP). The accumulation of high levels of the toxic lipid oxidation product 4-HHE may endanger microglia, whose role is complex, and likely to be a combination of positive and negative influences on the survival of neurons. Microglia provide an essential role in the support of neurons, and can provide a neuroprotective role. A study has shown a significant decrease in survival and an increase in disease progression which was positively correlated with the reduction in microglia and astrocytes in G93A-SOD1 mice [53]. There is much evidence that suggests a complex and changing role of glial cells such as the microglia during the evolution of the disease: thus, in the early stage of the disease microglia can have an anti-inflammatory and neuroprotective function (e.g. defined as a M2 phenotype), which is gradually evolving into a neurotoxic M1 phenotype in end-stage disease [54], [55]. Increased oxidative stress (increased formation of 4-HHE is a reflection of this oxidative stress) could have a critical role in this switch from protection to toxicity. Interestingly, imbalances in cellular antioxidant systems may be a particularly negative factor in the evolution of the disease against the G93A SOD1 mutation [56].

In summary, the predominant effects of dietary EPA in this animal model of ALS are damaging rather than neuroprotective. These observations are comparable to the disappointing and somewhat unexpected results reported with other potentially neuroprotective compounds or strategies, such as lithium, methylene blue or caloric restriction [20], [57], [58]. Therefore, the data highlight the need to encourage extreme caution for individuals with ALS (and especially in the context of SOD1 mutation carriers) in the use of dietary supplements of long chain omega-3 fatty acids such as EPA. As these negative observations have important implications for patients, a similar extensive study with DHA is required, in our view. Our results suggest that there is a distinct risk that prolonged exposure to preparations highly enriched in EPA could accelerate disease progression.

## Supporting Information

Figure S1 G93A-SOD1 129Sv mice with dietary EPA at the symptomatic stage of the disease do not have a significantly different development of the disease compared to animals on the control diet. (A) Consumption of food, (B) The body weight of mice, (C) Rotarod and (D) hindlimb extension reflex, and (E) grip strength were all not affected by dietary EPA compared to control diet.
(TIF)

Figure S2 G93A-SOD1 129Sv mice with dietary EPA at the pre-symptomatic stage of the disease have a non-significant exacerbation of disease phenotype compared to animals on the control diet. (A) survival (B) disease duration, and (C) disease onset were all affected by dietary EPA of 300 mg/kg/day compared to control diet.
(TIF)
