# Correction: The Omega-3 Fatty Acid Eicosapentaenoic Acid Accelerates Disease Progression in a Model of Amyotrophic Lateral Sclerosis

**DOI:** 10.1371/journal.pone.0307246

**Published:** 2024-07-11

**Authors:** Ping K. Yip, Chiara Pizzasegola, Stacy Gladman, Maria Luigia Biggio, Marianna Marino, Maduka Jayasinghe, Farhan Ullah, Simon C. Dyall, Andrea Malaspina, Caterina Bendotti, Adina Michael-Titus

In Table 1, there are errors in the numerical values presented. Please see the correct Table 1 here.

**Table 1:** Oral administration of EPA increases plasma EPA levels in G93A-SOD1 mice. Mice administered with either a single bolus dose of EPA (300 mg/kg, p.o.) or fed with an EPA enriched diet (300 mg/kg/day) for 7 days had higher levels of EPA and DHA in their plasma than mice fed on the control diet.

					Fold change	
Mouse strain	Fatty acid (% of total)	Control diet	EPA gavage	EPA diet	EPA gavage	EPA diet
C57BL6 mice	EPA	0.3±0.1	1.9±0.2*	2.0±0.1*	+6.3	+6.7
	DHA	3.1±0.9	5.3±0.4*	6.8±0.6*	+1.7	+2.2
	DHA/EPA	10.9±2.5	2.8±0.4*	3.4±0.4*	-3.9	-3.2
	Total n-3 PUFA	4.0±1.0	8.4±0.6*	9.6±0.5*	+2.1	+2.4
	Total n-6 PUFA	45.0±2.0	44.2±2.3	43.4±1.8	+1.0	+1.0
	Total PUFA	49.0±2.9	52.6±2.5	53.1±2.2	+1.1	+1.1
129Sv mice	EPA	0.4±0.1	1.5±0.5*	2.3±0.3*	+3.8	+7.7
	DHA	3.7±1.3	4.9±1.3*	6.3±0.7*	+1.3	+1.7
	DHA/EPA	9.0±2.4	3.6±1.6*	2.8±0.2*	-2.5	-3.2
	Total n-3 PUFA	4.8±1.6	7.3±1.4*	9.3±1.0	+1.5	+1.9
	Total n-6 PUFA	46.5±3.7	48.5±1.7	46.8±0.5	+1.0	+1.0
	Total PUFA	51.3±4.5	55.8±2.7	56.1±1.3	+1.1	+1.1

In Increased plasma EPA content after oral gavage or dietary supplementation with EPA subsection of Results, there is an error in the second sentence of the first paragraph. The correct sentence is: The dietary intake of EPA for 7 days by G93A-SOD1 C57BL/6 mice or 129Sv mice increased the levels of EPA by 6.7 or 7.7 fold, respectively, when compared to the control diet (Table 1).
