# Supplementary figures and images for: The Omega-3 Fatty Acid Eicosapentaenoic Acid Accelerates Disease Progression in a Model of Amyotrophic Lateral Sclerosis
Source: PLoS One. 2013 Apr 19;8(4):e61626. doi: 10.1371/journal.pone.0061626 (PMC3631166; doi:10.1371/journal.pone.0061626)

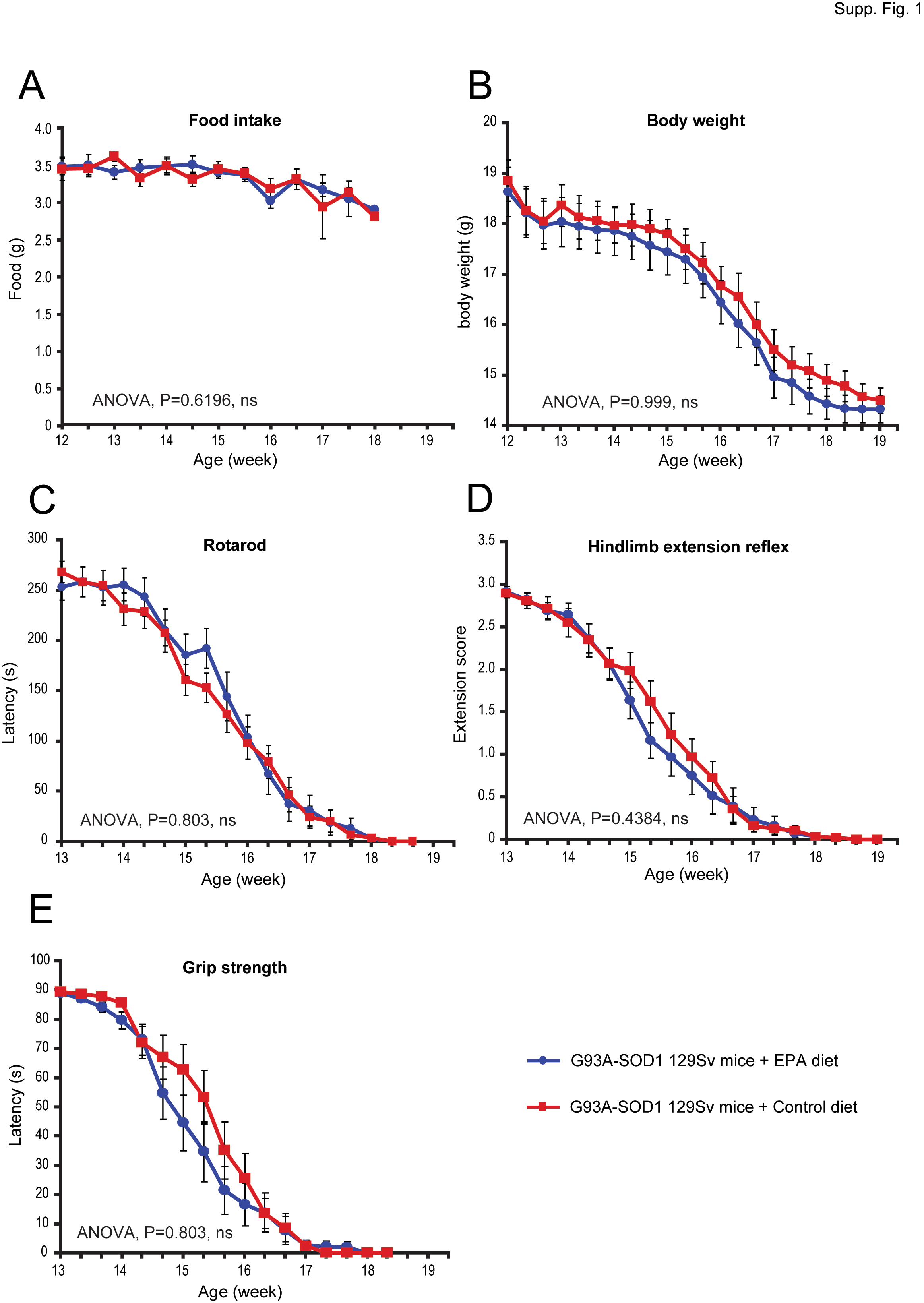

Supplement: Figure S1 — G93A-SOD1 129Sv mice with dietary EPA at the symptomatic stage of the disease do not have a significantly different development of the disease compared to animals on the control diet. (A) Consumption of food, (B) The body weight of mice, (C) Rotarod and (D) hindlimb extension reflex, and (E) grip strength were all not affected by dietary EPA compared to control diet. (TIF) [file pone.0061626.s001.tif]

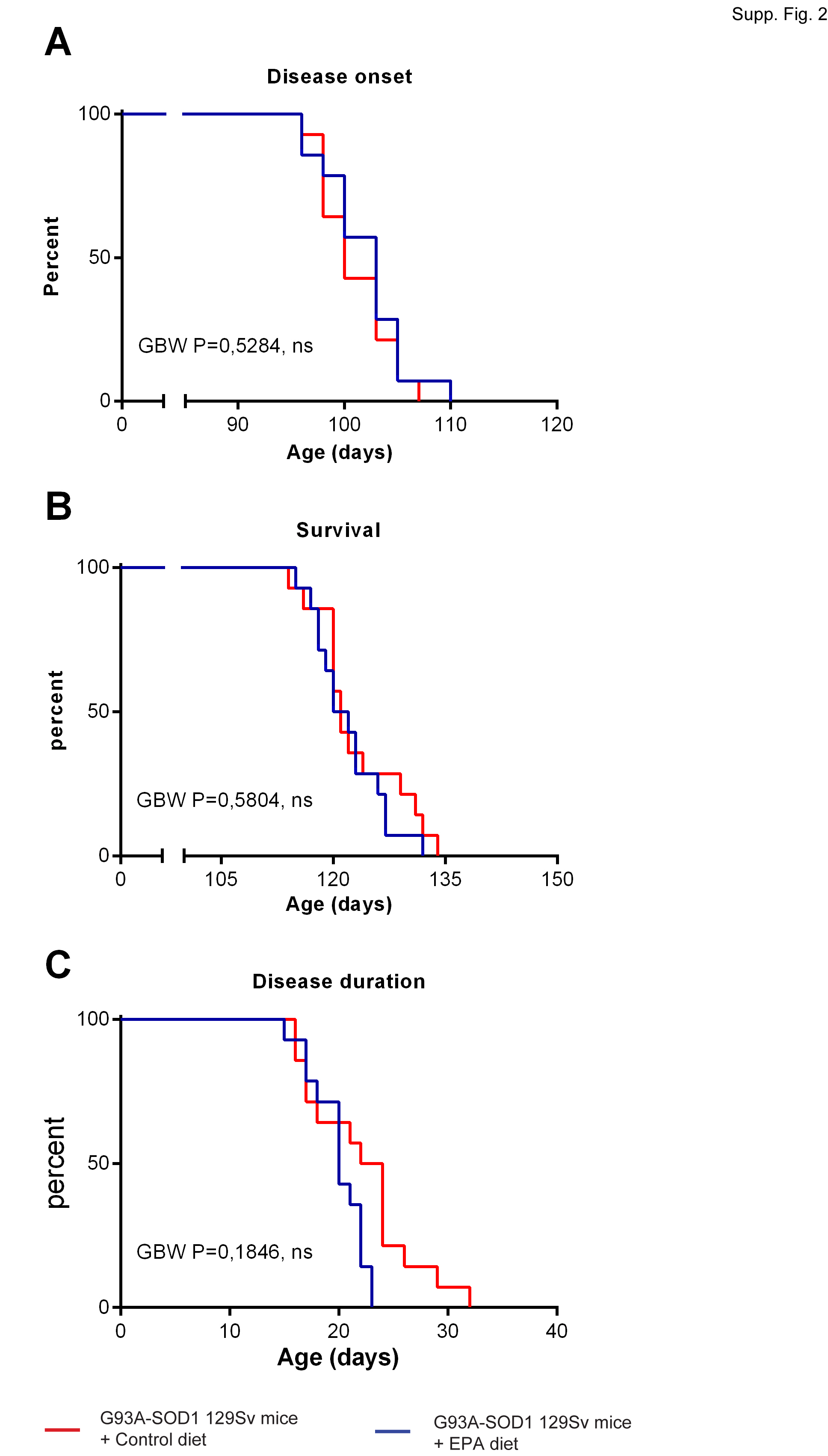

Supplement: Figure S2 — G93A-SOD1 129Sv mice with dietary EPA at the pre-symptomatic stage of the disease have a non-significant exacerbation of disease phenotype compared to animals on the control diet. (A) survival (B) disease duration, and (C) disease onset were all affected by dietary EPA of 300 mg/kg/day compared to control diet. (TIF) [file pone.0061626.s002.tif]
